# Sjögren’s Syndrome Complicated With Sarcoidosis With a Repetitive, Prolonged, Non-productive Cough

**DOI:** 10.7759/cureus.22827

**Published:** 2022-03-03

**Authors:** Takahito Nakamura, Takashi Watari, Shiro Ohshima, Utae Katsushima, Shigeo Muro

**Affiliations:** 1 Respiratory Medicine, Hoshigaoka Medical Center, Osaka, JPN; 2 General Medicine Center, Shimane University Hospital, Izumo, JPN; 3 Hospital Medicine, University of Michigan, MI, USA; 4 Rheumatology and Allergology, National Hospital Organization Osaka Minami Medical Center, Osaka, JPN; 5 Rehabilitation, Kansai Medical University Hospital, Osaka, JPN; 6 Respiratory Medicine, Nara Medical University, Nara, JPN

**Keywords:** gum test, prolonged cough, sicca symptom, sjögren's syndrome, sarcoidosis

## Abstract

Sjögren's syndrome and sarcoidosis are systemic immune-mediated diseases of unresolved pathogenesis, with dry cough being a symptom of both diseases. Due to the low pre-test probability of the diseases, they are not considered in the first differential diagnosis of prolonged non-productive cough. We report the case of a 33-year-old woman presenting with an intermittent, non-productive cough, diagnosed with Sjögren’s syndrome coexisting with sarcoidosis.

## Introduction

Sarcoidosis and Sjögren's syndrome are systemic immune-mediated diseases of unresolved pathogenesis, with dry cough being a symptom of both diseases. However, they are not considered in the first differential diagnosis of prolonged non-productive cough. Here we report the case of a 33-year-old woman presenting with an intermittent, non-productive cough, diagnosed with Sjögren’s syndrome coexisting with sarcoidosis.

## Case presentation

A 33-year-old Japanese woman without significant medical history presented to our hospital with a six-week period of intermittent, non-productive cough. Fourteen days before her admission to the hospital, she complained to her general practitioners about a persistent cough and chest pain while coughing. She was prescribed cough medicine and an analgesic, but her symptoms did not improve. Two days before visiting our hospital, the patient presented to the general practitioners again. They took a chest radiograph in anteroposterior view, which showed bilateral hilar enlargement; therefore, the doctors referred her to our hospital. The patient had no past medical history and was not taking any regular medicine. She worked as a car mechanic and used a preventative mask for dust and had no history of smoking, travel, or any apparent contact with sick people.

Upon examination, she appeared to be mildly ill with a persistent dry cough. Her vital signs were as follows: temperature, 36.6°C; blood pressure, 128/72 mmHg; heart rate, 80 beats/minute; respiratory rate, 20 breaths/minute; and oxygen saturation, 97% without supplemental oxygen. Examination of the oropharynx showed no pharyngeal edema or exudate other than mild dryness of the mucous membrane. Her neck was supple. Cardiovascular examination showed no abnormalities. Her lungs were clear to auscultation, and there was no evidence of superficial lymph node swelling. During the examination at our outpatient clinic, the patient complained of photophobia and blurred vision.

Methods and procedures

**Figure 1:**
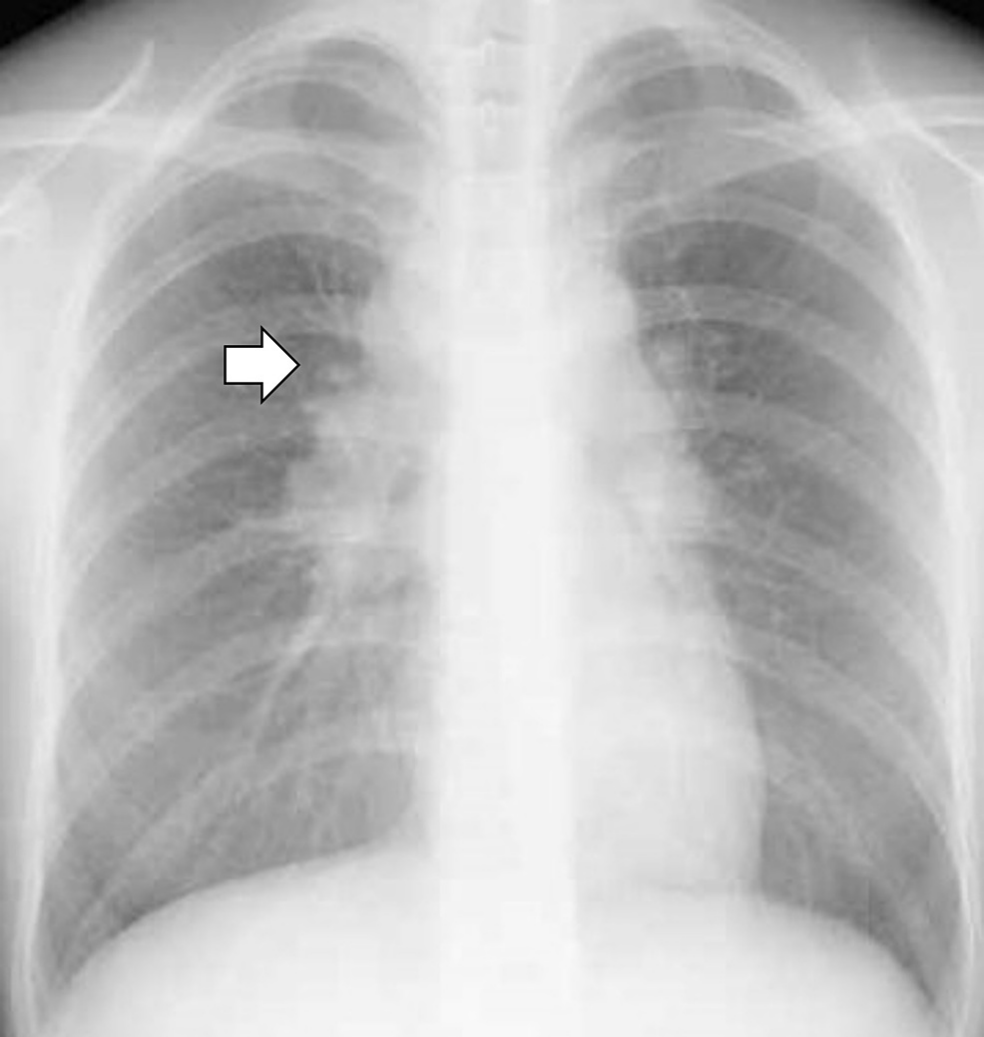
Chest radiography revealing bilateral hilar enlargement at the first visit.

**Figure 2:**
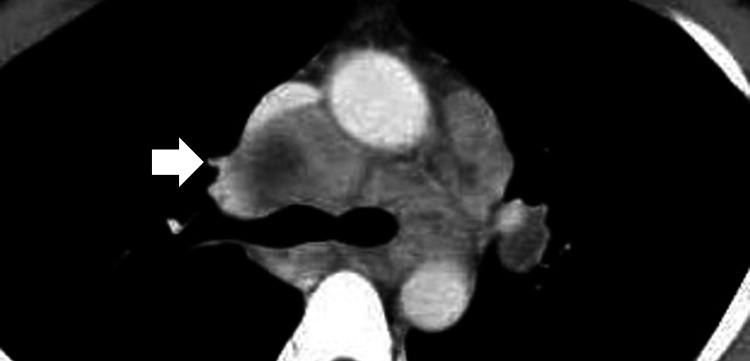
Computed tomography scanning of the chest revealing bilateral hilar and longitudinal lymphadenopathy at the first visit.

Chest radiography (Figure 1) revealed bilateral hilar enlargement. Computed tomography (CT) imaging of the chest (Figure 2) showed bilateral hilar and longitudinal lymphadenopathy without abnormality in the lung parenchyma such as interstitial pneumonia. Sputum cultures for bacteria and acid-fast bacilli were all negative. Blood examination revealed an elevation in soluble interleukin-2 receptor and angiotensin-converting enzyme levels to 3680 U/mL and 34.4 IU/L, respectively. Other blood examination results were normal. The electrocardiogram and echocardiography results were normal.

**Figure 3:**
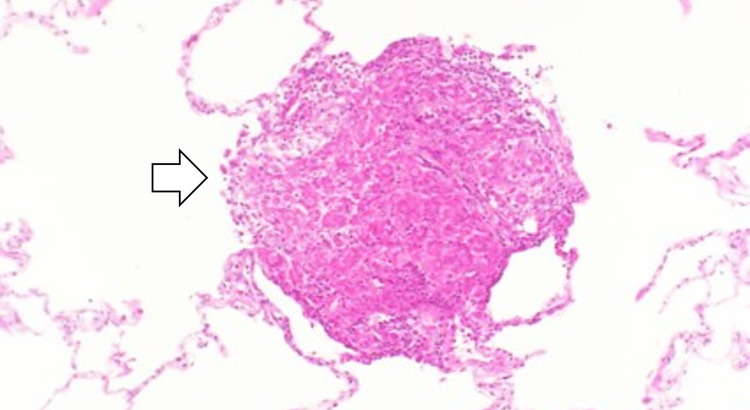
A transbronchial lung biopsy specimen from the patient’s lung parenchyma showing non-caseating epithelioid granuloma.

For further evaluation, we performed a bronchoscopy. There was no abnormality in the airway mucosa. Bronchoalveolar lavage fluid showed that the proportion of lymphocytes was 81%, and the ratio of CD4 to CD8 was 2.0, compatible with sarcoidosis. A transbronchial lung biopsy specimen from the patient’s lung parenchyma showed non-caseating epithelioid granuloma (Figure 3). We did not detect pathogens such as mycobacterium and mycosis from these specimens.

Additionally, the patient’s complaint of photophobia and blurred vision was diagnosed as uveitis by an ophthalmologist. Scintigraphy revealed high uptake of gallium-67 in the hilar and longitudinal lymph nodes, lacrimal glands, and parotid glands. Considering these results, we diagnosed her with systemic sarcoidosis.

For chronic dry cough, a gum test is usually done as a screening test to check for sicca symptoms, even in patients without dry mouth symptoms. In this patient, the gum test result revealed 7 mL of saliva secretion in 10 minutes, suggesting dry mouth. The patient had no dental caries or erosion of the mucosa; therefore, we followed up without medication. Additionally, the test for the anti-Ro/SSA autoantibodies was positive. Based on these results, we ordered dynamic salivary gland scintigraphy of 99mTc-sodium pertechnetate with lemon juice stimulation, revealing the hypofunction of salivary glands. Though we did not perform Schirmer's test in this patient, we diagnosed the patient with coexisting Sjögren's syndrome, according to the 1999 revised Japanese classification criteria.　

An antitussive drug (dextromethorphan 30 mg, three times a day) and a bronchodilator were administered to the patient without the use of inhaled steroids and antibiotics. The ophthalmologist prescribed localized steroid therapy, leading to the gradual improvement of uveitis.

**Figure 4:**
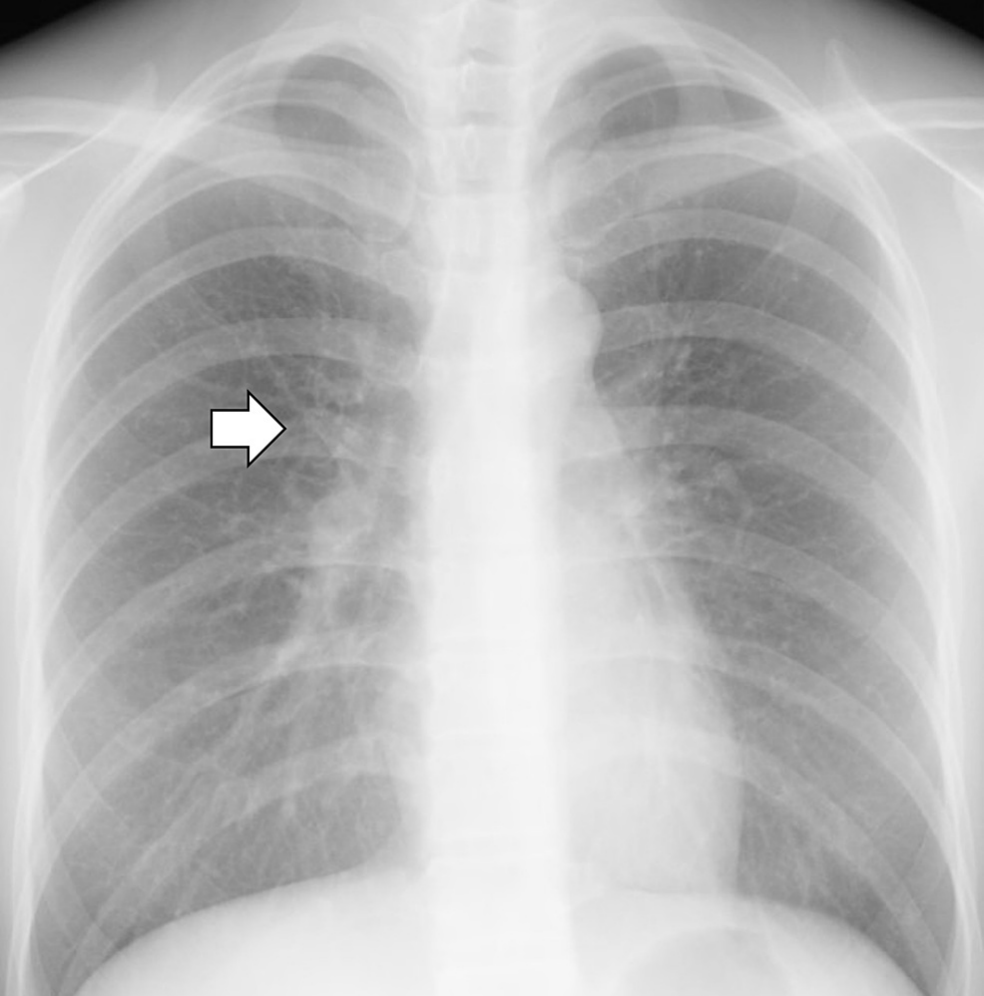
Chest radiography revealing a remarkable improvement of bilateral hilar enlargement

**Figure 5:**
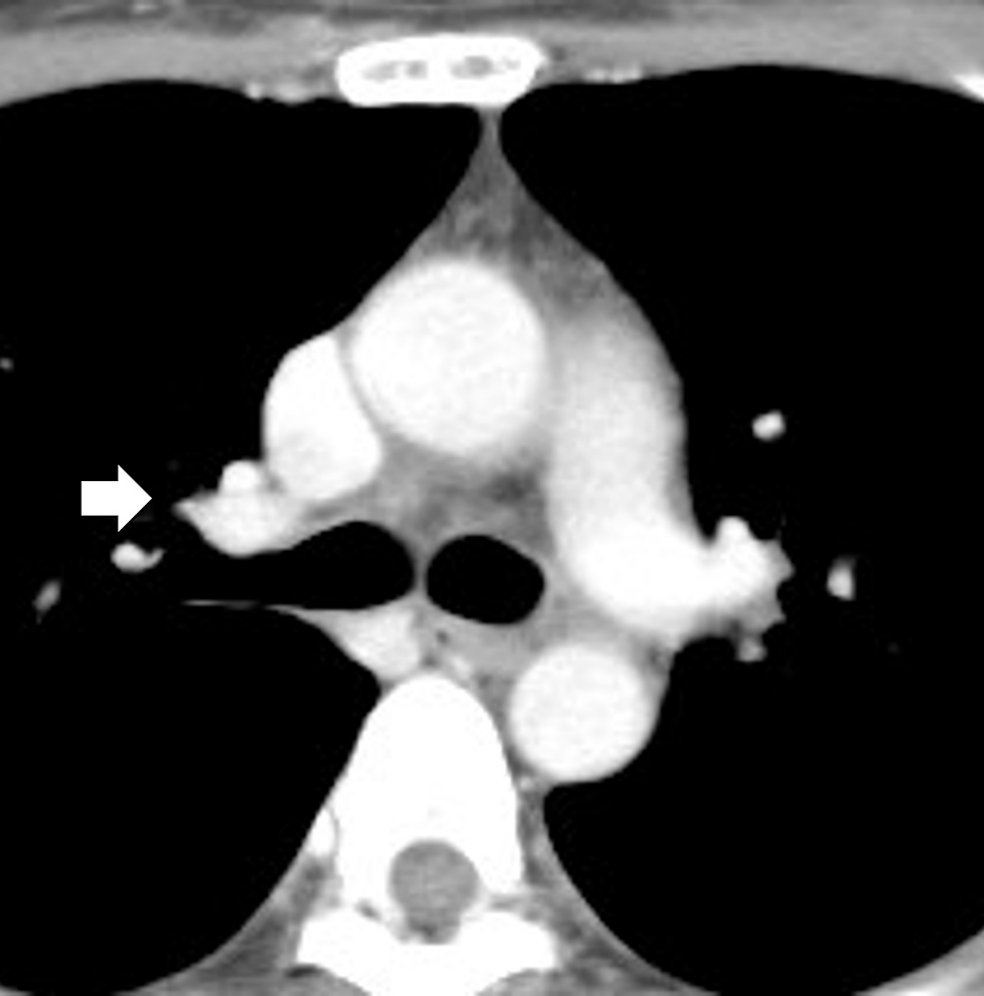
Computed tomography scanning of the chest revealing a remarkable improvement of hilar and longitudinal lymphadenopathy 13 months after the first visit

We carefully followed up with the patient, considering the possibility of lymphoproliferative diseases such as malignant lymphoma. Two months after her first visit, both chest CT scans and laboratory test results revealed an improvement. Seven months later, the patient recovered from her symptoms without using a bronchodilator. Thirteen months later, radiological findings of chest radiography (Figure 4) and CT (Figure 5) revealed a remarkable improvement of hilar and longitudinal lymphadenopathy.

Twenty-four months later, sicca symptoms improved as compared with the gum test at the initial visit. However, 32 months later, sicca symptoms such as dryness of mouth and intermittent non-productive cough recurred. The gum test revealed a decrease in saliva production to 3 ml per 10 minutes. However, the laboratory data such as serum angiotensin-converting enzyme (ACE) level, sIL2r, and radiological findings were within the normal range, suggesting no aggravation of sarcoidosis. The symptoms improved after administering inhaled steroids for symptom relief.

## Discussion

We reported a case of Sjögren's syndrome complicated with sarcoidosis, initially presenting as a prolonged non-productive cough. Sarcoidosis is a multiorgan disease of unknown etiology characterized by the accumulation of lymphocytes, mononuclear phagocytes, and noncaseating granulomas in the involved tissues. [1] Sjögren's syndrome is a chronic, multiorgan, autoimmune disease exhibiting lacrimal and salivary gland inflammation, leading to hypofunction of secretion such as dryness of the eyes and mouth. [2] The association between sarcoidosis and Sjögren's syndrome has been previously reported. [3] A report from Taiwan in 2017, states that Sjögren's syndrome was significantly associated with sarcoidosis (adjusted odds ratio, 11.6; 95% CI, 4.36-31.0). [4]

Dry cough occurs as a symptom of both sarcoidosis and Sjögren's syndrome. [5,6] We sometimes note chronic cough in patients with sarcoidosis. Increased cough sensitivity is associated with pathophysiology. [6] Of the 80 patients with newly diagnosed Sjögren's syndrome, 30 (37.5%) had a dry chronic cough, 14 (17%) of whom had been hospitalized for chronic cough. [7] Surprisingly, they also reported that patients with chronic cough were diagnosed 24 months later than those without chronic cough. [7] Sarcoidosis and Sjögren's syndrome are unlikely to be considered in the first differential diagnosis of prolonged non-productive cough. If the patient does not complain of sicca symptoms, the diagnosis of Sjögren's syndrome might initially be delayed. Thus, the patient might be misdiagnosed with other diseases such as cough variant asthma. The association between sarcoidosis and other autoimmune diseases has recently been reported, and attention should be paid to the complication of autoimmune diseases when treating sarcoidosis. There may be insufficient recognition that chronic cough is a symptom of sarcoidosis or Sjögren's syndrome. In addition, there may be insufficient recognition of sarcoidosis, Sjögren's syndrome, and their possible association as a differential diagnosis for chronic cough. Important implications of this case report are that Sjögren's syndrome and sarcoidosis should be considered in patients with chronic cough of uncertain cause and the gum test should be used as a screening test because both diseases may present with Sicca symptoms such as the dry mouth. [8-10]

In clinical practice for chronic cough, we can inexpensively screen for sicca symptoms by performing a gum test. [7,8] Although it is difficult to distinguish clearly whether the cause was sarcoidosis or Sjögren's syndrome at the time of recurrent cough in this patient, based on the results of the gum test and the stable condition of sarcoidosis, the sicca symptoms due to Sjögren's syndrome may have more impact on the recurrent cough. In patients first diagnosed with Sjögren's syndrome or sarcoidosis, we should take the co-occurrence of both diseases into account as a differential diagnosis. Knowing whether a patient has Sjögren's syndrome or sarcoidosis makes it easier for us to decide what points we should pay attention to during follow-ups.

## Conclusions

Sarcoidosis and Sjögren's syndrome should be considered in the differential diagnosis of non-productive coughs. In clinical practice for chronic cough, we can inexpensively screen for sicca symptoms by performing a gum test, leading to detection of diseases such as Sjögren's syndrome and sarcoidosis. If sarcoidosis or Sjögren's syndrome is suspected, the possibility of both should be considered as a differential diagnosis.
